# D-BRAIN: Anatomically Accurate Simulated Diffusion MRI Brain Data

**DOI:** 10.1371/journal.pone.0149778

**Published:** 2016-03-01

**Authors:** Daniele Perrone, Ben Jeurissen, Jan Aelterman, Timo Roine, Jan Sijbers, Aleksandra Pizurica, Alexander Leemans, Wilfried Philips

**Affiliations:** 1 iMinds - IPI - TELIN, Ghent University, Ghent, Belgium; 2 iMinds - Vision Lab, Department of Physics, University of Antwerp, Antwerp, Belgium; 3 Image Sciences Institute, University Medical Center Utrecht, Utrecht, The Netherlands; University of Minnesota, UNITED STATES

## Abstract

Diffusion Weighted (DW) MRI allows for the non-invasive study of water diffusion inside living tissues. As such, it is useful for the investigation of human brain white matter (WM) connectivity in vivo through fiber tractography (FT) algorithms. Many DW-MRI tailored restoration techniques and FT algorithms have been developed. However, it is not clear how accurately these methods reproduce the WM bundle characteristics in real-world conditions, such as in the presence of noise, partial volume effect, and a limited spatial and angular resolution. The difficulty lies in the lack of a realistic brain phantom on the one hand, and a sufficiently accurate way of modeling the acquisition-related degradation on the other. This paper proposes a software phantom that approximates a human brain to a high degree of realism and that can incorporate complex brain-like structural features. We refer to it as a Diffusion BRAIN (D-BRAIN) phantom. Also, we propose an accurate model of a (DW) MRI acquisition protocol to allow for validation of methods in realistic conditions with data imperfections. The phantom model simulates anatomical and diffusion properties for multiple brain tissue components, and can serve as a ground-truth to evaluate FT algorithms, among others. The simulation of the acquisition process allows one to include noise, partial volume effects, and limited spatial and angular resolution in the images. In this way, the effect of image artifacts on, for instance, fiber tractography can be investigated with great detail. The proposed framework enables reliable and quantitative evaluation of DW-MR image processing and FT algorithms at the level of large-scale WM structures. The effect of noise levels and other data characteristics on cortico-cortical connectivity and tractography-based grey matter parcellation can be investigated as well.

## Introduction

One of the biggest challenges the neuroscientific community has been facing is the investigation of the living brain white matter (WM). The advent of diffusion weighted (DW) MRI in the 1980s [1] has made this investigation feasible. However, many technical limitations affect the estimation of WM brain features. Acquisition artifacts due to mechanical vibrations [2], noise [3], and other imperfections degrade the estimation of WM features significantly. Given the limited DW MRI scan time for clinical protocols, spatial resolution is often sacrificed to reduce scan time in clinical protocols. This leads to image degradations such as the partial volume effect [4–6] and the Gibbs Ringing effect [7, 8]. Patient head motion during acquisition [9, 10] often causes additional image degradation. In diffusion tensor imaging (DTI, [11]), given the lower diffusion gradient strength used, the influence of imperfect acquisition is less problematic. However, the diffusion tensor is not suitable to model WM diffusivity at high diffusion gradient attenuation, nor to detect multiple bundles in crossing fibers regions. In fact at the voxel level, the WM diffusivity profile is geometrically very complex, which has resulted in the development of many model-based [12] or model-free [13–16] methods. Consequently, estimating this complex profile in the presence of the aforementioned artifacts is problematic. A recent publication [17] extensively reviews these methods.

There are many fiber tractography methods that aim to reconstruct the WM fiber connectivity in great detail [5, 18, 19]. However, intra voxel diffusion estimation is influenced by clinical acquisition strategies, e.g., the number of acquired DW directions [20]. Additionally, some of the streamlines estimated by such fiber tractography (FT) methods may be actually false positives, whereas smaller WM bundles may not be detected and tracked [21]. As a result, the inter voxel streamline estimates produced by such (FT) methods are inaccurate. Moreover, FT techniques are subject to integration [22, 23], termination [24], and over-fitting [25] errors.

Developing brain FT techniques which mitigate those limitations remains a big challenge, as it is a proper evaluation of tractography. For instance, in [26], a comparison of FT algorithms is presented based on human data acquired with a single b-value of 1000 s/mm^2^. Although the results show good agreement between FT techniques, in the absence of a ground truth data set, such results cannot be conclusive in an absolute sense.

So far, a number of different approaches have been developed to construct a gold-standard diffusion MRI phantom which can be categorized as software phantoms, hardware phantoms, and biological phantoms. The focus of this paper is on creating software phantom data. A first reason is that a software phantom allows one to test the susceptibility of methods to acquisition-related degradation. This type of research is impossible with a biological [27–30] or a hardware phantom [31–37] as in these cases the phantom images are acquired by an MR scanner in the first place. Indeed, MR acquisition artifacts can not be simulated unambiguously as the phantom data itself is not artifact-free to begin with. Instead, artifacts can be simulated with frameworks like BrainWeb [38]. Additionally, manufacturing a hardware phantom of adequate complexity can be a very challenging task, whereas the microstructural organization and the ground-truth connectivity pattern of the biological case are generally unknown. Prior knowledge of such characteristics is important as recently, neuroscientists have started to use graph theory [39–41] as a new tool for analyzing human brain (network) disorders [42–52] and differences in human brains [53, 54]. In neuroscience, connectome features are usually compared by means of connectivity matrices (CMs). CMs are a compact and structured way that allows to interpret brain connectomes as graphs. Parcellated (segmented) grey matter (GM) regions represent vertices, while estimated streamlines connecting them are considered as edges of a network. These studies are very promising in terms of gaining insight into certain psychopathologies, and would benefit substantially from phantom data, which are more realistic in terms of human brain structures, which have a perfectly known ground truth connectivity, and which allow for a more accurate simulation of MR acquisition in all its imperfections.

Tournier et al. [55] introduced phantoms based on apparent diffusion coefficient (ADC) and fractional anisotropy (FA), while Leemans et al. simulated the cerebellum of a starling by using realistic mean diffusivity (MD) and FA values [56]. Van Hecke et al. [57] simulated brain-like DTI atlases. Close et al. [58] made available a phantom consisting of densely packed bundles of fibers featuring a more flexible geometrical complexity. In these works, however, the diffusion attenuation is modeled with a tensor or a mixture of tensors but without including geometrical properties of the brain itself, therefore partly limiting the use of the phantoms for evaluating general processing methods.

In recent work, the intra-voxel diffusivity has been modeled with Monte-Carlo approaches like the ones described in [29, 59]. Such methods try to mimic a realistic biological environment and are very demanding in terms of computing power. However, realistic brain-like WM bundles geometries are not included in these frameworks either.

Recently, the Fiberfox phantom was proposed to tackle some of these limitations [60]. Fiber strands are drawn in 3D by a user, the method then builds a phantom from this input. The acquisition protocol model included in the Fiberfox phantom is realistic, and it uses diffusivity models estimated from real data. It was shown that the approach can produce a realistic replica of the FiberCup phantom [61], mimic the corticospinal tract [62], and produce realistic brain-like diffusion MRI data in context of the ISMRM 2015 tractography challenge, using 26 manually delineated WM bundles. This manual operation could be impractical, time-consuming, and requires expert neuroanatomical knowledge. Although existing software phantoms are generally very flexible, most of them are currently not realistic in terms of spatial geometry, microstructure modeling, WM bundle organization, or acquisition protocol in a unified framework. As such, studies that make use of these methods may not be suitable for providing reliable results in human connectomics [39, 63]. The goal of this paper is to complement and extend existing methods. We take a Fiberfox-like approach, and we extend on it. In our approach, the manually selected subset of bundles is replaced by a complete set of fiber data obtained using state-of-the-art, high-quality and high-resolution data and reconstruction methods, to obtain a phantom that features a more realistic level of complexity. This is obtained without the need of manual intervention.

In this work, we present a simulation framework to construct ground-truth diffusion MRI data that resemble the architecture of a human brain geometrically, microstructurally, and spatially in a single model, while mimicking data characteristics of a real acquisition. The resulting ground-truth simulation phantom basically represents a diffusion MRI brain, coined D-BRAIN, and is composed of DW MRI data obtained from estimated WM tracts, is embedded in a human brain-like anatomy with a realistic level of complexity, and includes several brain tissue types. Specifically, it includes diffusion features based on microstructural models with tissue characteristics derived from real data. A FT result obtained from a high quality dataset of a real brain, and having a much higher complexity than existing software phantoms, provides the WM geometric information for the model. The acquisition steps are also carefully simulated to mimic realistic acquisition protocols. Our framework provides realism in terms of acquisition and different brain tissues, while at the same time, it closely approximates WM microstructure and complex fiber bundle organization, approaching a complete and realistic whole brain acquisition simulation. It is very flexible and allows for a wide variety of parameters to be specified: the intrinsic tissue parameters and the MR scanner parameter settings for the simulated acquisition. As such, we propose a framework that allows one to investigate DW MRI related algorithms in a realistic setting, and to analyze their results at the brain connectivity level. Preliminary results of this work have been presented at the Joint Annual Meeting ISMRM-ESMRMB 2014 [64].

## Materials and Methods

The goal of the presented work is to obtain a brain model that is rich in both anatomical and diffusion-related details.

To this end, a connectome and tissue volume fraction maps are first estimated from a high-quality DW MRI scan of a brain. Afterwards, these estimates are used as input to insert a brain-like complexity in the phantom. In combination with the simulated MR acquisition protocol, the proposed framework provides a realistic full-brain DW MR data sampling.

**Fig 1:**
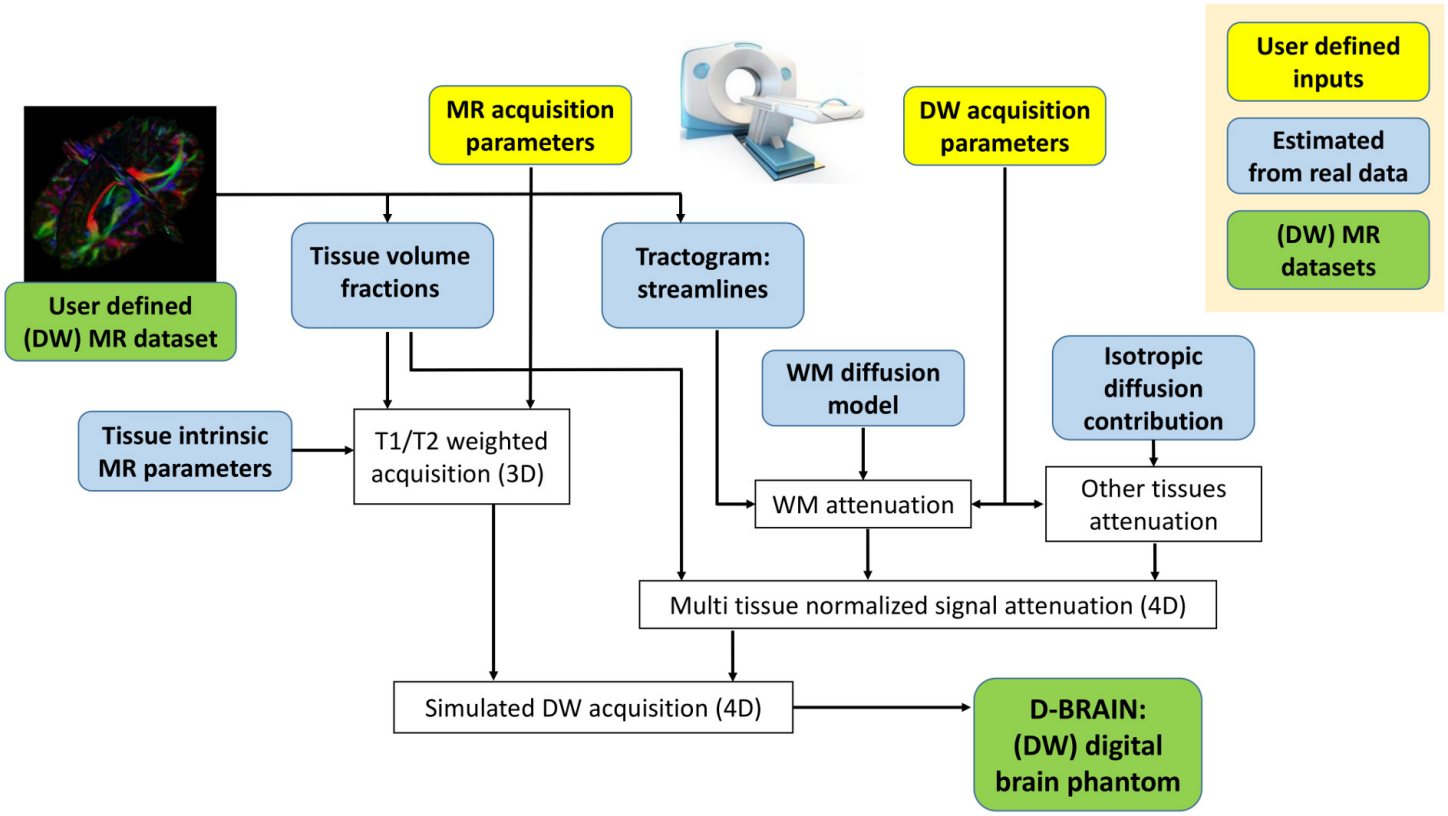
Brain like phantom creation. Proposed pipeline: from realistic inputs and a simulated DW MRI scanner to brain-like D-BRAIN data. The tissue volume fractions have a resolution of 0.7 × 0.7 × 0.7 mm^3^, streamlines step-size is 0.7 mm.

Fig 1 shows a schematic overview of the phantom simulation framework, whereas details will be elucidated in present section. Specifically, in subsection Definition of anatomy and diffusion architecture, we explain how the anatomical and diffusion structures that are used as input are extracted from a DW MRI data set of the human brain. In the second subsection, we present the tissue parameters that were used to give realistic features to the phantom DW data. In subsection Diffusion modeling, we show how the tissue-dependent diffusion attenuation was modeled and finally, in subsection Whole brain acquisition modeling, we explain how the whole-brain simulated acquisition protocol is obtained. Lastly, in subsection Connectome estimation, we illustrate the tools and parameters for a connectivity experiment we performed on D-BRAIN data.

### Definition of anatomy and diffusion architecture

The complex anatomical and WM structures that we incorporate in our phantom are estimated based on volume fractions of the main tissue types and the related fiber tract pathways reconstructed from high—quality DW MRI data.

Data was acquired on a customised Siemens Magnetom Skyra 3T MRI system equipped with a 32-channel receiver head coil as part of the Human Connectome Project [65]. Diffusion weightings of b = 0, 1000, 2000, 3000 s/mm^2^ were applied in 18, 90, 90 and 90 directions, respectively. In addition, all images were acquired with reversed phase encoding, for the purpose of EPI distortion correction. Other imaging parameters were: TR/TE: 5520/89.5 ms, voxel size: 1.25 × 1.25 × 1.25 mm^3^, matrix: 145 × 145, slices: 174 and NEX: 1. T1-weighted structural images were acquired, to aid identification of the different tissue types, with a spatial resolution of 0.7 × 0.7 × 0.7 mm^3^. The detail about the DW EPI images preprocessing pipeline is documented in the paper of Jeurissen et al. [66]. The resulting DW images are aligned geometrically to each other, and to the corresponding structural data.

The four tissue types (CSF, cortical GM (CGM), deep GM (DGM) and WM) were segmented on the structural image using the state-of-the-art framework outlined in Smith et al. [24]. The approach combines several tools from the FMRIB Software Library (FSL), to obtain a reliable partial volume fraction map for all four tissue types. The estimated tissue volume fraction (VF) maps have a resolution of 0.7 × 0.7 × 0.7 mm^3^. In the following sections, we will refer to it as “input VF.”

In our pipeline, WM geometric information is obtained via the whole-brain, probabilistic fibre tracking as implemented in MRtrix (https://github.com/MRtrix3/mrtrix3, [67]) using the 2nd order integration over fODFs (iFOD2) [23]. The fODFs are estimated using the multi-tissue, multi-shell CSD approach developed by Jeurissen [66]. We used Anatomically Constrained Tractography (ACT, [24]) with GM-WM interface seeding based on the four tissue types segmentation from above, to ensure anatomically plausible fibre reconstructions. The final tractogram is composed by 5 × 10^7^ streamlines with a mean step size of 0.7 mm. The subvoxel resolution allows to minimize voxel-quantization errors in the tract orientation, such that these fibers smoothly cross the voxel borders following the main diffusivity orientations, just like it is expected from the real WM connections. In addition, this set of streamlines was further reduced to 5 × 10^6^ streamlines using “spherical-deconvolution informed filtering of tractograms” (SIFT) [25], to account for seeding biases and improve the correspondence of the Track Density [68] and the underlying Apparent Fibre Density [69] obtained with multi-tissue, multi-shell CSD. In the following sections, we will refer to it as the “input connectome.”

These inputs correspond to the blocks “Tissue volume fractions” and “Tractogram” in Fig 1.

### Tissues intrinsic parameters

Anatomical MR image contrast is determined by intrinsic parameters such as the tissue proton density (PD) and the relaxations times (spin-lattice and spin-spin, commonly named as T1 and T2, respectively). These relaxation times depend on the scanner field strength. In our framework, we allow one to choose among the constants already available in POSSUM [70] to simulate the use of a 1.5-T scanner and a 3.0-T scanner (Table 1). Our method supports extensions to higher field strength acquisitions, given the appropriate scanner/tissue parameters. In Fig 1, these inputs correspond to the block “Tissue intrinsic MR parameters.”

**Table 1:** D-BRAIN sensitivity to magnetic fields.

1.5T scanner	WM	GM	CSF	3T scanner	WM	GM	CSF
T1 [ms]	500	833	2569	T1 [ms]	832	1331	3700
T2 [ms]	70	83	329	T2 [ms]	44	51	500
PD	0.77	0.86	1	PD	0.77	0.86	1

Scanner dependent tissue parameters.

In DW imaging, the MR scanner uses an additional gradient to image the direction-dependent attenuation caused by the diffusion of water molecules inside the brain tissues. For the (normalized) diffusion attenuation signal, a different analytical model was used to compute the response provided by each tissue subject to the diffusion-sensitizing gradient pulse. In this paper, the intra voxel diffusion models we used are an isotropic “tensor” for CSF and CGM, while a “zeppelincylinder” was chosen for WM, because of its accuracy in describing the properties of WM [71]. DGM was modeled as a mixture of CGM and WM. We used intrinsic parameters for these models that were inferred from in vivo human brain [72, 73]. In the following section, letting *j* be the index of the tissue types, we will generically refer to the diffusion related intrinsic parameter set as **p**_*j*_. These are reported below:

- WM: cylinder (59% of the signal), diffusivity = 1.49 × 10^−9^ m^2^ s^−1^, radius = 4.8 × 10^−6^ m; zeppelin (41% of the signal) diffusivity, parallel = 1.49 × 10^−9^ m^2^ s^−1^, perpendicular = 0.72 × 10^−9^ m^2^ s^−1^;
- CGM: isotropic tensor, constant FA = 0 and MD = 0.83 × 10^−9^ m^2^ s^−1^;
- CSF: isotropic tensor, constant FA = 0 and MD = 3.19 × 10^−9^ m^2^ s^−1^;
- DGM: mixture of WM (20% of the signal) and CGM (80% of the signal).

In Fig 1, these correspond to the blocks “WM diffusion model”and “Isotropic diffusion contributions.”

### Diffusion modeling

We can compactly write the generic, multi-tissue diffusion-weighted signal as:

$$\begin{array}{c} \frac{S_{k}\left(r\right)}{S_{0}\left(r\right)}=\sum_{j=1}^{P}v_{j}\left(r\right)A_{j,k}. \end{array}$$ (1)

In this equation, *S*_*k*_(**r**) and *S*_0_(**r**) are the intensities of the DW MR signal acquired along a specific direction *k* and the anatomical reference signal, respectively. The factors *v*_*j*_(**r**) are the volume fractions of each tissue *j* and are stored in the “input VF” (see subsection Definition of anatomy and diffusion architecture). Depending on the spatial position considered, there could be up to *P* tissues in a voxel **r**.

The specific, tissue-dependent normalized diffusion weighted attenuation *A*_*j*, *k*_ (corresponding to the block “Multi tissue signal attenuation” in Fig 1) depends on other diffusion sequence (MR scanner) parameters **q**. In our method **q** is composed of the gradient duration δ, time between two pulses Δ, gyromagnetic ratio γ, and diffusion gradient strength G. In addition to the parameters **q**, the normalized diffusion weighted signal also depends on the acquisition direction *k* (optimized as proposed in [74]) and on the tissue-dependent diffusion parameters **p**_*j*_ as defined in subsection Tissues intrinsic parameters.

Note that the definition of *A*_*j*, *k*_ is not unique. In fact, a distinction is needed between different brain tissues. For GM and CSF, the normalized diffusion weighted attenuation has an isotropic diffusion pattern that can be written as:

$$\begin{array}{c} A_{j,k}=A_{k}\left(p_{j},q\right). \end{array}$$ (2)

In Fig 1, the equation above corresponds to the block “Isotropic diffusion contributions.” For the WM tissue, the geometric information from the “input connectome” has to be included as well. The WM normalized diffusion attenuation model requires an additional input, that is, the orientation of the WM streamlines. In this work, we first assign each streamline segment, *i*, of the “input connectome” to a specific voxel, depending on the spatial position considered. There could be up to *N*(**r**) streamline segments in a voxel **r**. Afterwards, we compute the WM normalized DW attenuation, depending on the direction **d**(*i*) of each of the connectome segments *i* in a voxel as:

$$\begin{array}{c} A_{j,k}=\frac{1}{N(r)}\sum_{i=1}^{N(r)}A_{k}\left(p_{j},q,d\left(i\right)\right), \end{array}$$ (3)

which corresponds to the block “WM attenuation” of Fig 1.

### Whole brain acquisition modeling

In this work, the “simulated acquisition” step carefully accounts for the MR physics. MRI is based on the measurements of the net magnetization of hydrogen nuclei in a volume, subject to a static magnetic field *B*_0_. Each tissue *j* has its own proton density *PD*_*j*_ (which is obtained as described in subsection Tissues intrinsic parameters), such that the tissue-related magnetization *M*_*j*_ can be written as

$$M_{j}\approx PD_{j}{\left(\frac{\gamma \text{h}}{2\pi }\right)}^{2}\frac{1}{4K_{b}T_{s}}B_{0},$$ (4)

where γ is the hydrogen gyromagnetic ratio, *h* is Planck’s constant, *K*_*b*_ is Boltzmann’s constant and *T*_*s*_ is the sample temperature. Depending on the specific composition, each tissue returns to the equilibrium state after *T*1_*j*_ (spin-lattice) and *T*2_*j*_ (spin-spin) relaxation times (again, these parameters are obtained as described in subsection intrinsic parameters).

In case of a regular multislice 2D spin-echo (SE) sequence, *T*1_*j*_ and *T*2_*j*_ relate to the MR signal intensity with the following model:

$$\begin{array}{c} S_{0,j}=M_{j}\exp\left(-\frac{TE}{T2_{j}}\right)\left(1-\exp\left(-\frac{TR}{T1_{j}}\right)\right). \end{array}$$ (5)

Therefore, in this case, the *TE* and the *TR* are the acquisition parameters which can be used to maximise the contrast for specific tissue types.

Eq 5 applies to a single, specific tissue type. However, in reality, multiple tissue types—up to *P*—may make up the area represented by a voxel **r**, as specified from the “input VF” (see subsection Definition of anatomy and diffusion architecture). In this case, once the equation is evaluated for one tissue type *j*, the results are linearly combined using the factor *v*_*j*_(**r**) obtained from the “input VF”, reflecting the physics of the scanning process. That is,

$$\begin{array}{c} S_{0}\left(r\right)=\sum_{j=1}^{P}v_{j}\left(r\right)S_{0,j}. \end{array}$$ (6)

In Fig 1, this step corresponds to the block “T1/T2 weighted acquisition.” The “acquired” brain images are simulated at the maximally available resolution, 0.7 × 0.7 × 0.7 mm^3^. This is a key strength of the presented framework, because in clinical DW MRI protocols the standard resolution is much lower. In addition, the spatial resolution can be adjusted by mimicking the way the MR scanner operates. In case of a regular multislice 2D SE sequence, for instance, the resolution along the axial dimension can be reduced by modeling the slice selection pulse as an ideal rectangular function. Afterwards, each image k-space is downsampled and low-pass filtered. According to [75], scanners often use Fermi and Hamming filters, which are also included in our framework. For an even more realistic acquisition, noise can be added at k-space level before the smoothing step. In this way, we can introduce noise, partial volume effects, and truncation artifacts, therefore replicating the acquisition obtained with a real world MR machine. Further physiologically related data conditions, such as pulsation and subject motion artifacts, can be added as well as described previously [10, 76]. These features are included in the block “Simulated DW acquisition” in Fig 1.

To recap, our framework allows the user to choose extrinsic parameters such as the echo time (TE), and the repetition time (TR), together with the voxel size, the noise level, the diffusion attenuation strength, the gradient pulse duration δ, and the interval between the two of them, Δ, as well as the number of DW gradient directions, therefore approaching a regular DW MR acquisition and creating brain-like data as would be acquired with a real MRI acquisition protocol (see the “User defined inputs” blocks in Fig 1).

### Connectome estimation

The target of our paper is to create phantoms with brain-like complexity. To verify this complexity, it makes sense to study FT results in terms of connectivity matrices (CMs).

We therefore investigated several connectivity metrics widely used in the research area of connectomics, to assess their robustness with respect to noise and partial volume effect. Tensor based deterministic tractography was performed using ExploreDTI [77] on D-BRAIN data with different noise levels and different resolutions. 50 noisy realizations of D-BRAIN data were simulated for each noise level and two different voxel sizes, while diffusion tensors were estimated using the RESTORE method [78]. We extracted one fiber pathway per voxel, across different resolutions. FA for seeding and terminating a tract was set to 0.1 and the maximum curvature angle was set to 45. CGM and DGM were parcellated in 70 cortical and 12 subcortical regions using Freesurfer [79]. Connectivity matrices were estimated using ExploreDTI [77] and the Brain Connectivity Toolbox [63] was used for the connectivity metrics estimation.

## Results

In this section, we demonstrate the potential of the proposed framework, by showing its realism in terms of WM geometry and its versatility in terms of simulated acquisition. We first show how to simulate a D-BRAIN acquisition in subsection Simulated D-BRAIN acquisitions. Secondly, we illustrate some of the geometrical features inferred in the phantom (subsection D-BRAIN spatial and geometric features). Afterwards (subsection Effect of SNR, b-value, and resolution), we show the phantom sensitivity to different acquisition parameters and lastly, (subsection D-BRAIN for connectomics) we demonstrate the utility of the proposed phantom from a connectivity point-of-view.

### Simulated D-BRAIN acquisitions

Based on the inputs and the models explained in the sections above, we created different “simulated D-BRAIN acquisitions” with the (DW) MR scanner parameters reported below.

- Field strength = 3T;
- Resolution = 0.7 × 0.7 × 0.7 mm^3^, 1.4 × 1.4 × 1.4 mm^3^ and 2.1 × 2.1 × 2.1 mm^3^;
- TR / TE = 8800 / 57 ms;
- 6 b = 0 s mm^−2^ and 60 directions with a b-value of 1000 s mm^−2^, 2500 s mm^−2^ and 10000 s mm^−2^;
- Diffusion pulses duration δ = 12.9 ms;
- Interval between diffusion pulses Δ = 21.8 ms.

Phantom data sets used for the connectivity metrics estimation have the following parameters:

- 6 b = 0 s mm^−2^ and 60 directions with a b-value of 1000 s mm^−2^;
- Resolution = 1.4 × 1.4 × 1.4 mm^3^ and 2.1 × 2.1 × 2.1 mm^3^;
- SNR of 30, 25, 20, 15;
- 50 D-BRAIN datasets for each noise level.

We will use these data sets to assess the realism of the proposed phantom in the following subsections.

### D-BRAIN spatial and geometric features

We start our validation by visually investigating the images coming from the simulated diffusion MRI acquisitions, we examine the output tractogram and some biomarker-related properties.

**Fig 2:**
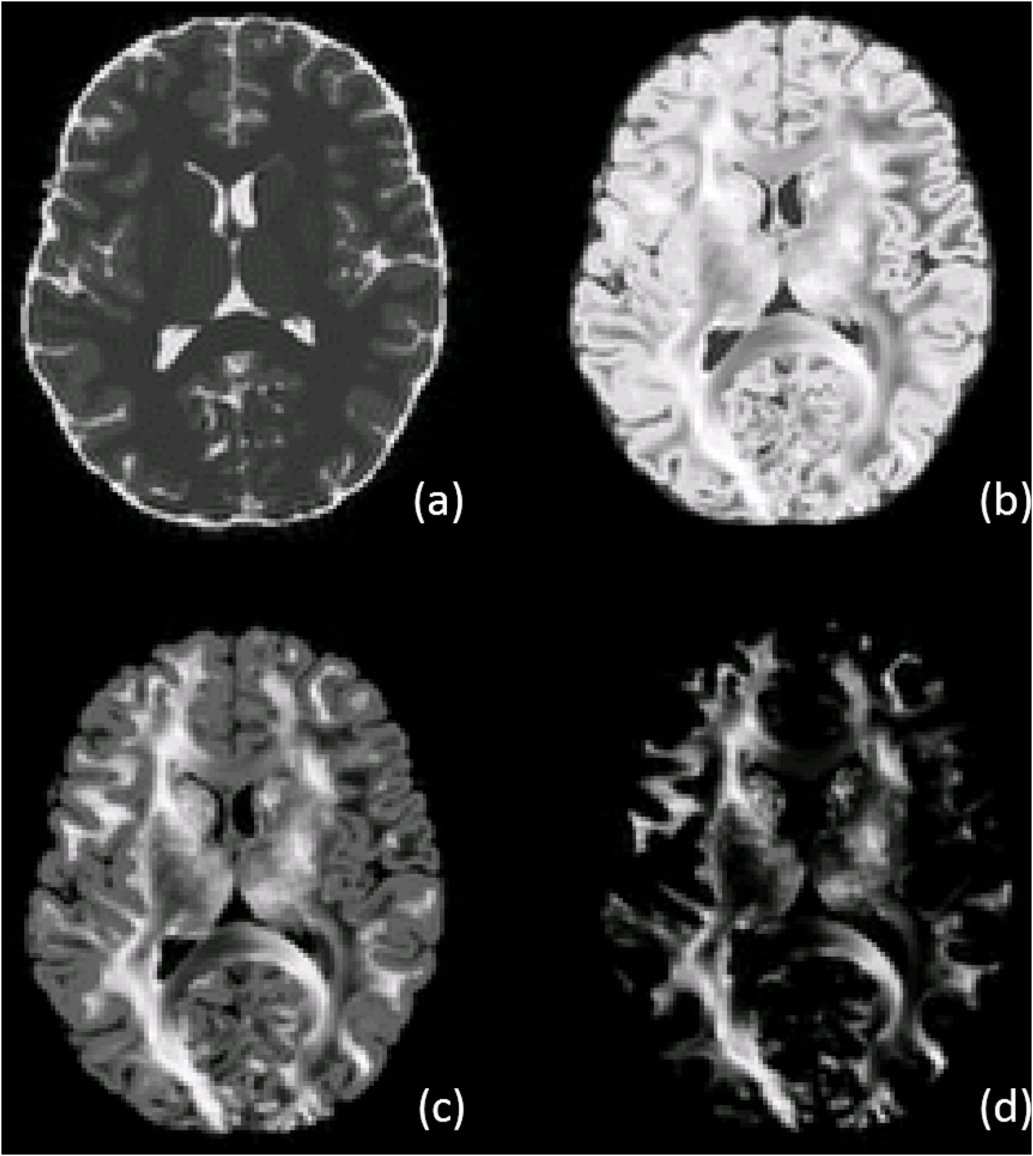
D-BRAIN. Anatomical MR image (a) and corresponding diffusion-weighted images for D-BRAIN data. B-values of 1000 s mm^−2^ (b), 2500 s mm^−2^ (c) and 10000 s mm^−2^ (d). For each picture, the intensity values have been optimized for visual purposes.

Fig 2 shows images of the phantom DW MRI data at a resolution of 1.4 × 1.4 × 1.4 mm^3^ for b = 0 s mm^−2^, b = 1000 s mm^−2^, b = 2500 s mm^−2^ and b = 10000 s mm^−2^, The anatomical image (a) shows that we are able to include realistically looking brain structures in the phantom dataset, notably in terms of WM, CGM, DGM and CSF, and acquisition-related artifacts like Gibbs ringing effect and partial voluming. Additionally, complex diffusivity features are seen in WM regions (b-d), dipendent on the simulated diffusion attenuation.

**Fig 3:**
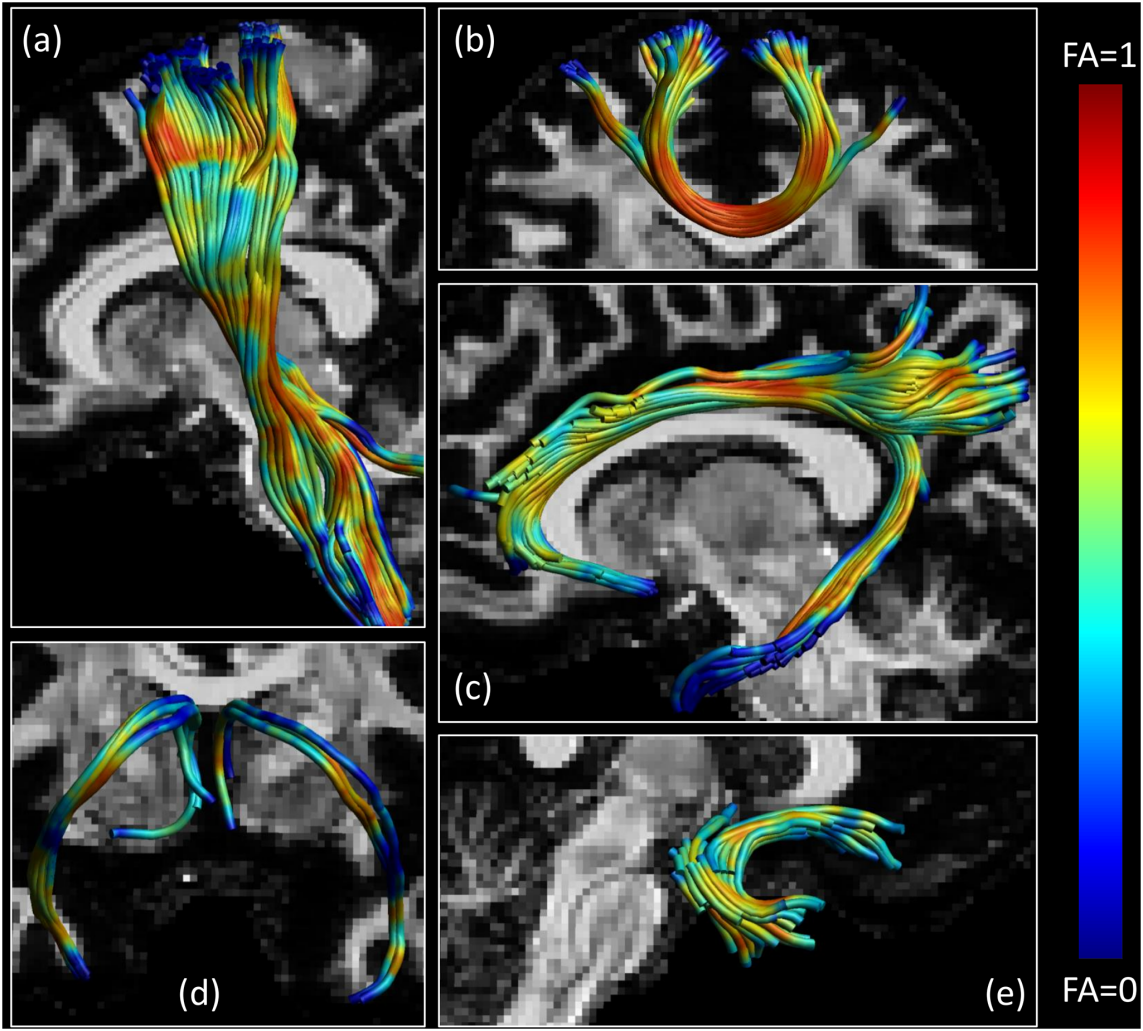
Phantom WM bundles tractography. A portion of the corticospinal tract (a), pathways of the forceps minor (b), tracts following the cingulum (c), streamlines belonging to the fornix (d), and part of the uncinate fasciculus (e).

**Fig 4:**
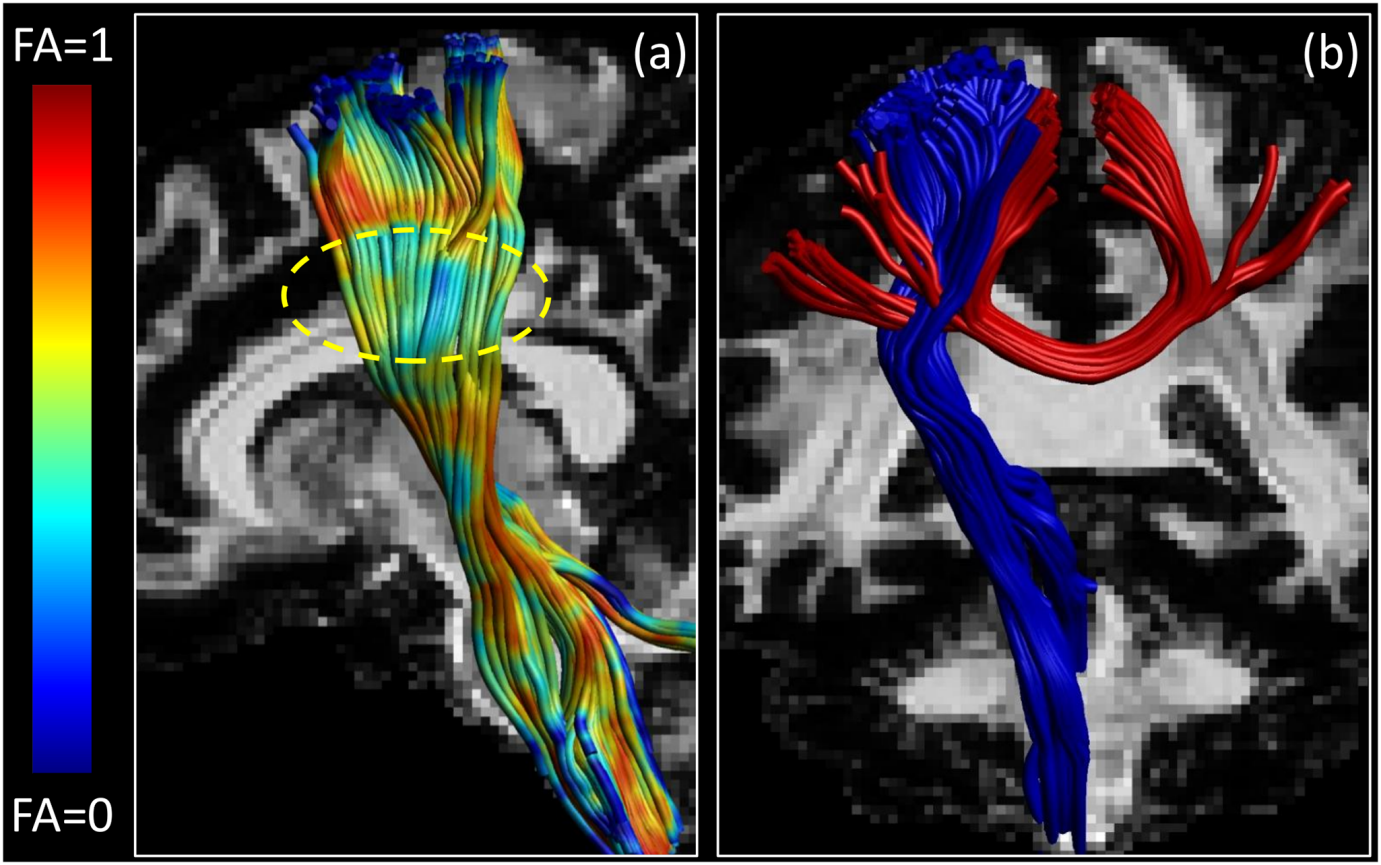
Phantom WM bundles tractography. The upper portion of the corticospinal tract (a) has a lower FA in the region highlighted because of the contribution of other fibers coming from the body of the corpus callosum. In (b), the crossing of the corticospinal tracts (blue) and the lateral projections of the corpus callosum (red) are clearly visible.

Deterministic CSD-based tractography was performed using ExploreDTI [77] on noiseless D-BRAIN data with voxel size of 1.4 × 1.4 × 1.4 mm^3^. Realistic streamlines are estimated (Fig 3), corresponding to the well-known pattern of the corticospinal tract (a), traversing the genu of the corpus callosum (b), following the cingulum bundle (c), the fornix (d), and the uncinate fasciculus (e). The tracts we show are color-encoded according to their FA: yellow areas reveal high FA, whereas red areas indicate low FA. In deep WM, the reason for a low FA is usually the presence of a region with crossing fibers. We find that the upper portion of the corticospinal tract shows a lower FA, because other tracts coming from the body of the corpus callosum traverse the same region, as elucidated in Fig 4.

### Effect of SNR, b-value, and resolution

We now demonstrate the use of our phantom by performing a study on the influence of some acquisition protocol parameters, i.e. the b-value, resolution, and SNR on biomarkers, such as FA and the fiber orientation distribution functions (fODF).

**Fig 5:**
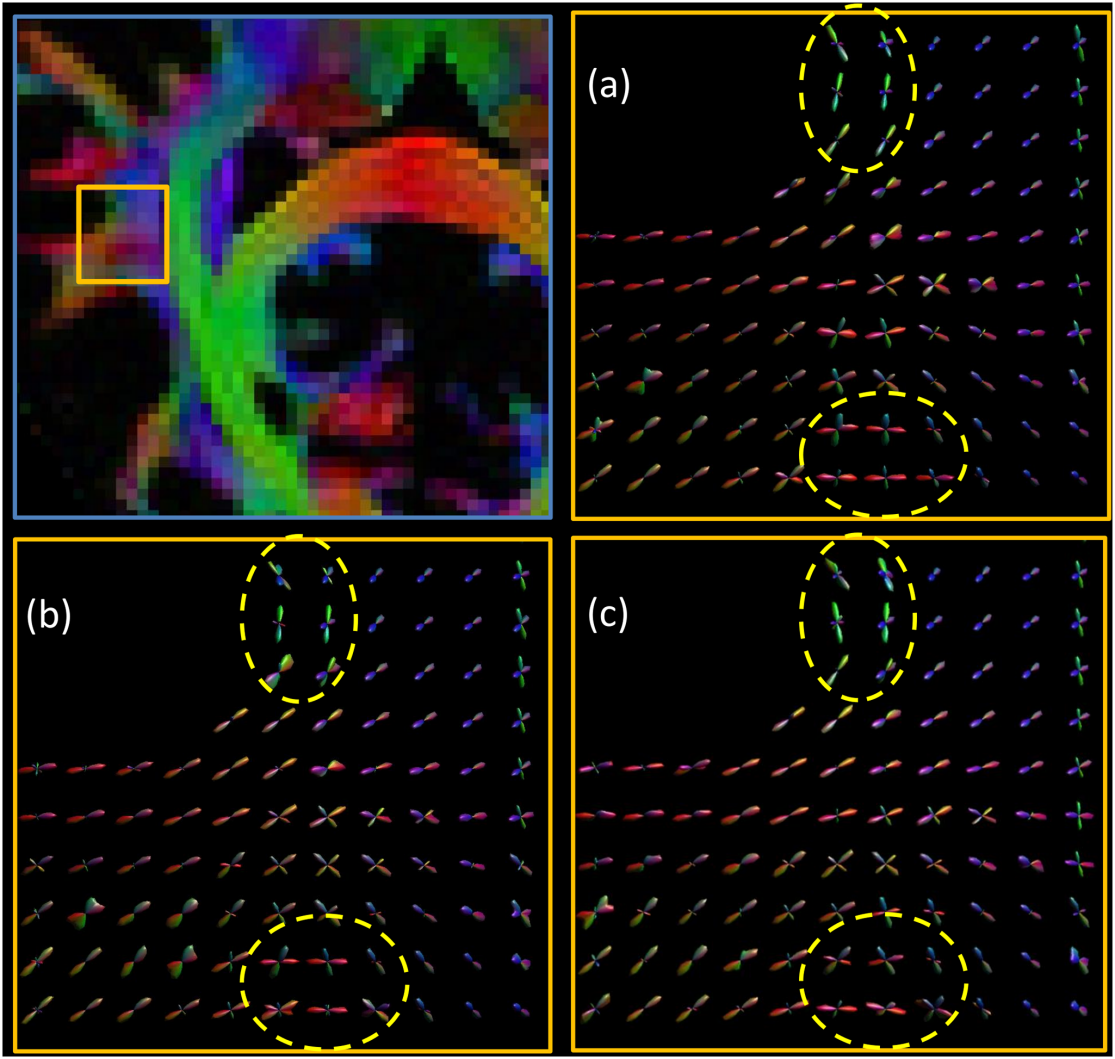
Estimated phantom fODF for different SNR. Yellow boxes highlight the effect of noise level. Top left: ROI. ROI magnification for: SNR = 30 (a), SNR = 20 (b), and SNR = 15 (c). B-value = 1000 s mm^2^.

**Fig 6:**
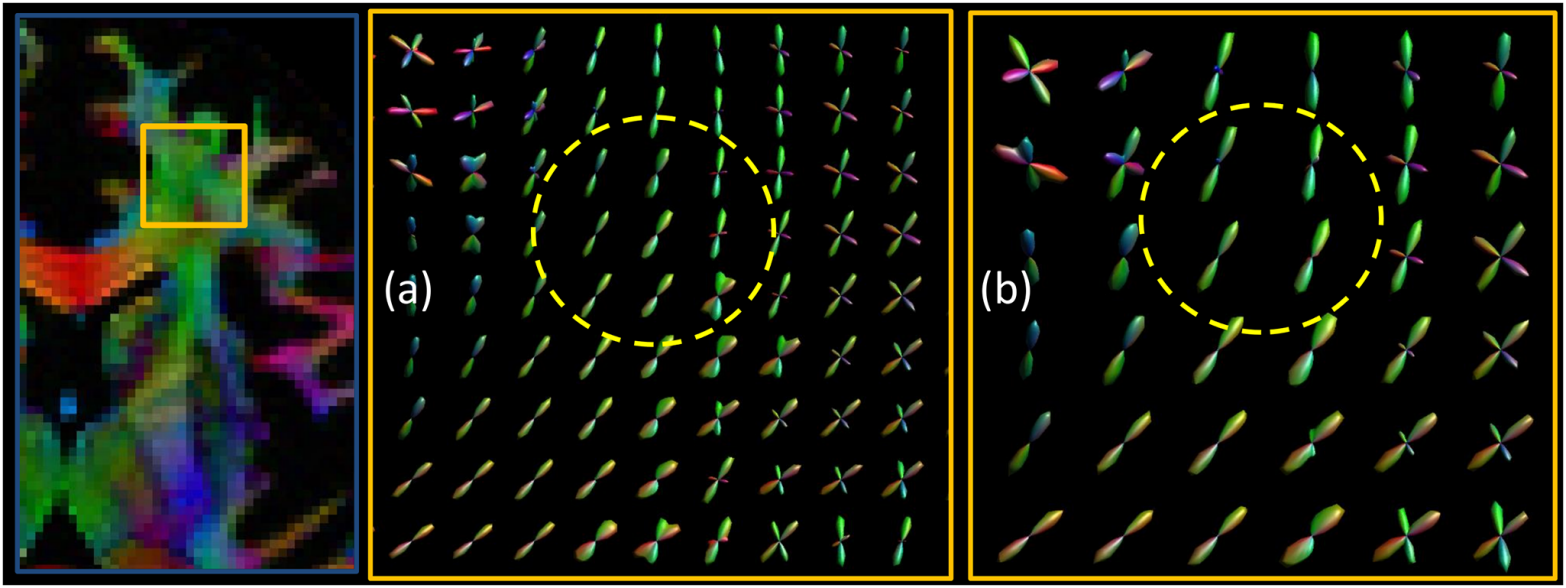
Estimated phantom fODF for different resolutions. Top left: ROI. ROI magnification for: voxel size = 1.4 × 1.4 × 1.4 mm^3^ (a), voxel size = 2.1 × 2.1 × 2.1 mm^3^ (b). SNR = inf.

**Fig 7:**
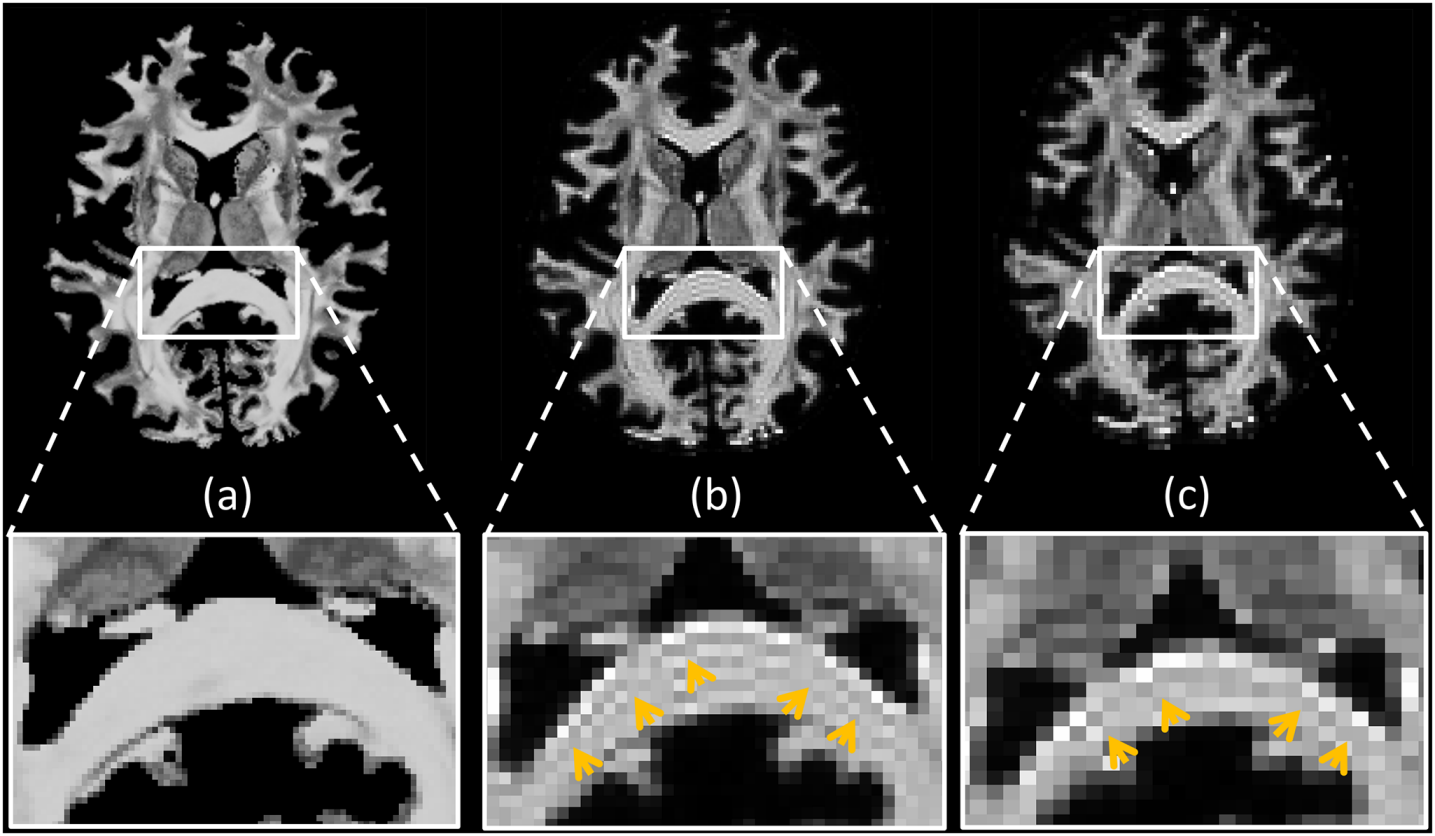
D-BRAIN FA maps before and after k-space downsampling. Voxel size is 0.7 × 0.7 × 0.7 mm^3^ (a), 1.4 × 1.4 × 1.4 mm^3^ (b) and 2.1 × 2.1 × 2.1 mm^3^ (c). Yellow arrows highlight the Gibbs ringing artifact.

**Fig 8:**
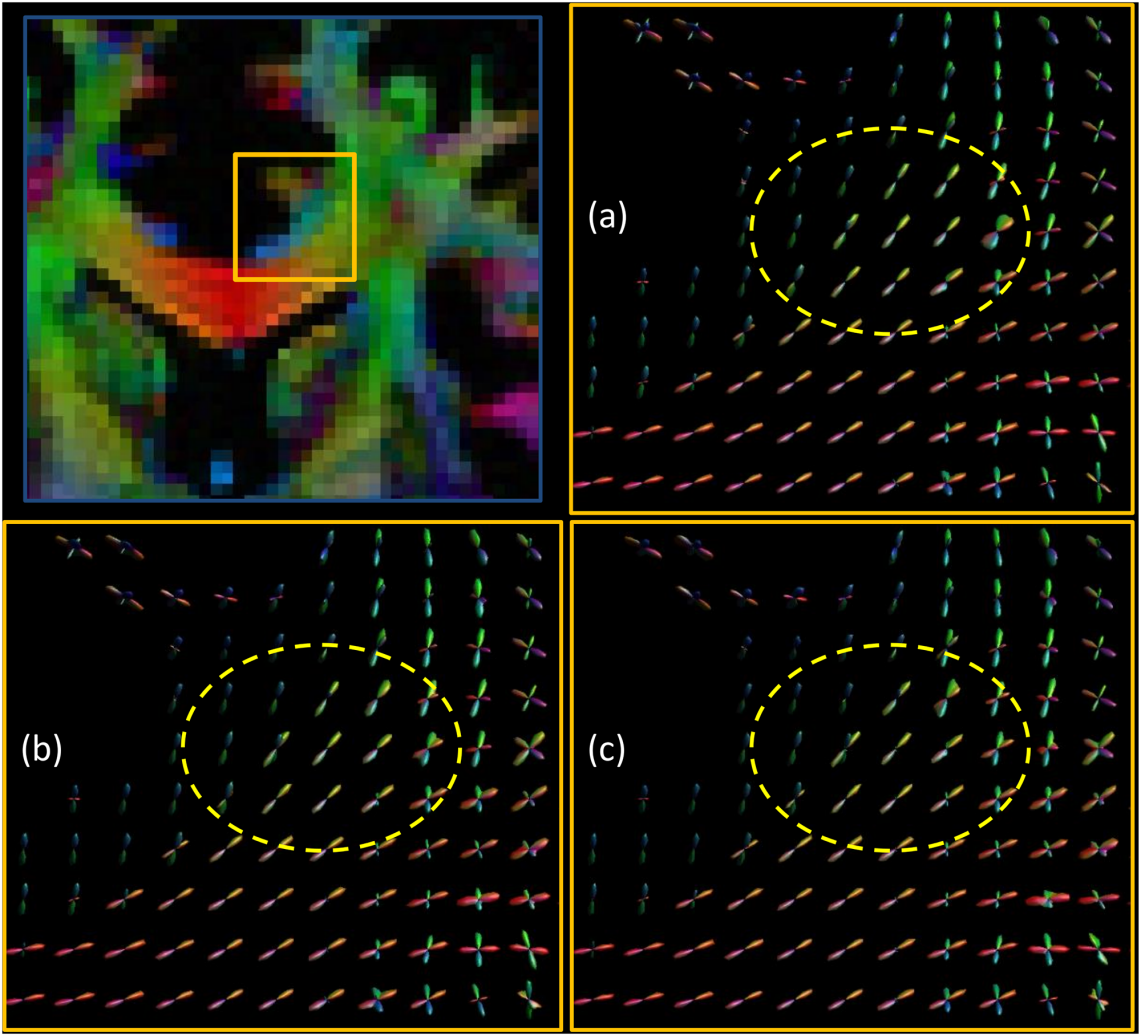
Estimated phantom fODF for different b-values. Yellow boxes highlight the effect of the b-value. Top left: ROI. ROI magnification for: b-value = 1000 s mm^2^ (a), b-value = 2500 s mm^2^ (b), b-value = 10000 s mm^2^ (c). SNR = inf.

In Fig 5 we investigated the behavior of phantom-derived fODFs with respect to noise. We observe that, as the noise increases, the variance of the CSD peaks becomes higher, therefore the estimated directions become less precise. In Fig 6, we compared D-BRAIN simulated scans with different resolutions. The effect of reduced resolution in a crossing fiber region on the estimated fODF can be appreciated from the enlarged regions. Introducing a lower resolution also results in another artifact, as shown in Fig 7, which shows FA maps with a ground-truth resolution of 0.7 × 0.7 × 0.7 mm^3^, 1.4 × 1.4 × 1.4 mm^3^ and 2.1 × 2.1 × 2.1 mm^3^. The distorted FA estimates indicated with the yellow arrows are manifestations of Gibbs ringing artifacts [7, 8] due to our accurate simulation of limited acquisition band-width. In Fig 8, the sensitivity of D-BRAIN with respect to the choice of b-value is demonstrated. Decreased precision and the occurrence of spurious fODF peaks can be observed for lower b-values which can be attributed to the lower angular diffusion contrast and the non-WM isotropic components that complicate the optimization for CSD [6, 33].

### D-BRAIN for connectomics

**Fig 9:**
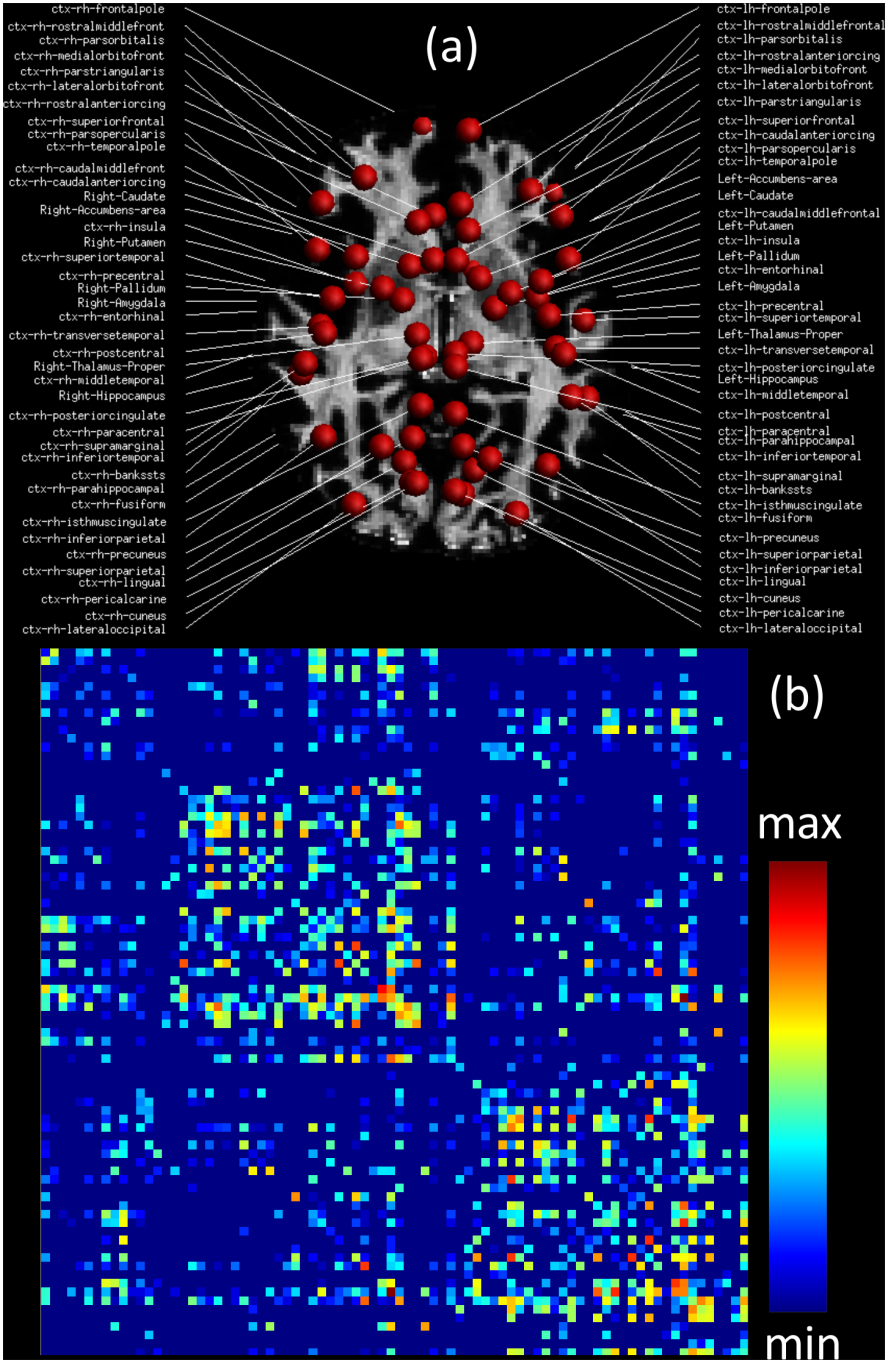
Connectivity analysis. The 82 GM parcels used as network nodes (a) superimposed to the FA map. In (b), the corresponding connectivity matrix built counting the number of streamlines connecting each couple of parcels (intensities displayed in logarithmic scale).

Lastly, we assessed the utility of the proposed phantom with a connectomics oriented comparison. In Fig 9, we show (a) the set of nodes used for the connectivity analysis, and (b) the CM corresponding to a tractogram estimated from noiseless D-BRAIN data at a resolution of 1.4 × 1.4 × 1.4 mm^3^ and b = 1000 s mm^−2^, whose intensities represent the number of estimated streamlines connecting each couple of nodes.

**Fig 10:**
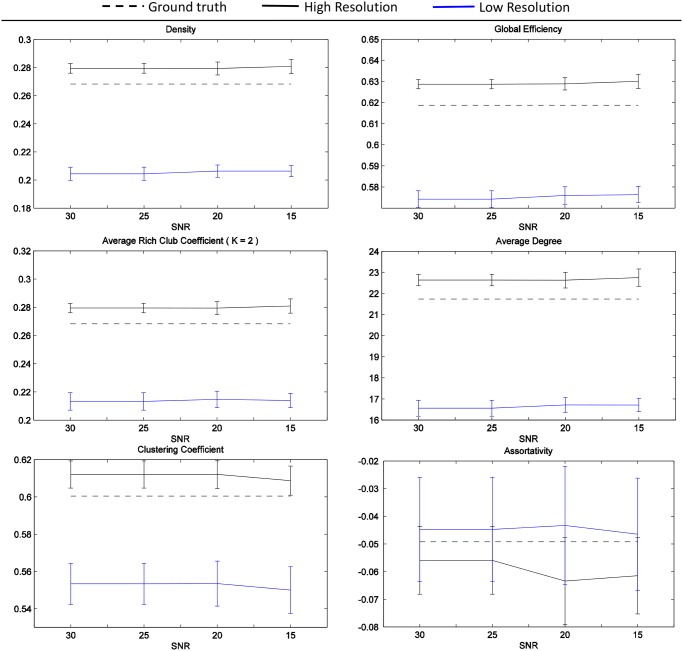
Estimated network connectivity measures. Variability across different SNR and resolutions of D-BRAIN data. Ground truth: from noiseless data whose voxel size is 1.4 × 1.4 × 1.4 mm^3^. High Resolution: from noisy data whose voxel size is 1.4 × 1.4 × 1.4 mm^3^. Low Resolution: from noisy data whose voxel size is 2.1 × 2.1 × 2.1 mm^3^.

Concerning the experiment assessing the variability of connectivity metrics, we show the results in Fig 10. The metrics have been derived from binary (unweighted) CMs, that have a value of 1 if there is at least one streamline connecting each couple of nodes and a value of 0 otherwise. Under these acquisition and analysis settings, we observe that the degradation introduced by partial voluming is in general larger than the bias introduced by noise. The network assortativity seems to be less sensitive.

Interestingly, the network degree, global efficiency, and clustering coefficient metrics estimated for the low resolution D-BRAIN data are in agreement with the ones reported in [80] that were computed from binary CMs estimated from real data acquired with a resolution and a diffusion weighted attenuation close to the one we simulated.

## Discussion

DW MRI data acquisition settings or tractography performance should ideally be studied with respect to phantom data with an adequate level of complexity. These considerations convinced us to include complex sets of streamlines coming from a real data set into our phantom and to use data driven analytical characterizations of the intra voxel diffusion models. Other phantoms have been extensively used as a test bench for FT algorithms comparison. An example is the Fibercup phantom [81]. However, it has been also criticized for its quite low FA that privileges a certain class of techniques [82], and, on top of that, it is geometrically much simpler than a human connectome, as many other software phantoms are.

In fact, we show that our framework is able to simulate realistic FA and fODF maps, and that tracking complex neural bundles in our phantom is possible. A good tractography method should be able to trace the bundles correctly, and the proposed method allows those kind of investigations, since it automatically includes a brain-like anatomy of high-level complexity. Additionally, a variety of DW MRI related problems can be investigated with brain-like D-BRAIN data in a realistic acquisition setting. The result of Fig 5, known from the literature, is retained within our framework and reflects the physical realism of the proposed phantom. In Fig 8, we show that a relistic relationship between the fODFs and the b-value is seen in regions where fibers are expected to cross in a complex way for our phantom, and at the WM-GM interface. This again emphasizes the level of realism of the proposed phantom. Also, Fig 6 shows that, by increasing the voxel size, the different compartment contributions cause finer WM structures gradually disappear from the fODF. This outcome is coherent with the established properties of WM features estimation via CSD. Because of our realistic acquisition protocol simulation, the proposed phantom and MR scanner simulation combination allows one to evaluate the robustness of methods with MR artifacts like partial voluming, angular resolution, angular contrast, and Gibbs ringing effect among others.

Segmentation of realistic cortical and subcortical structures based on their connectivity pattern was attempted in [83, 84]. However, it is not clear how these methods perform depending on acquisition parameters. Studying the efficacy of these techniques is now made easier with our method. As demonstrated in [85] for instance, the differentiation of specific bundles may depend on the spatial resolution: this needs a phantom of variable resolution. Many other possible acquisition scenarios can be simulated. In fact, a wide class of algorithms can be tested on D-BRAIN data, leading to conclusions that are coherent with established properties of real brains, since it includes brain-like spatial and geometric information and a complete simulation of the DW MRI acquisition process. Most importantly, we proposed a complete framework that simplifies the study of connectivity measures with respect to acquisition protocol parameters.

Lastly, we noticed that many connectivity-related measures from literature [63] require hard thresholding and binarization of CMs as a pre processing step. This helps to exclude false positive streamlines (that are therefore considered as noise) from analyses, and to reduce inter-data variability at the same time. However, if these streamlines are estimated by a FT algorithm that enforces a correspondance with the biological data, like SIFT does, this raw quantization may lead to a loss of useful information, as also hypothesized in [86]. More extensive studies on different realizations of our phantom may reveal the benefit of a different CM thresholding approach, if the target is to investigate the variability of graph theory related connectivity measures.

Our assumption about homogeneity within each brain tissue is a simplification; the phantom would become even more realistic if a greater number of tissue classes is included, like proposed in [87]. We tested the possibility to use a more complex model to approximate the GM diffusivity attenuation, the “astrocylinder” [71]. However, we found that it fits the GM diffusion attenuation across multiple b-values only if a biologically unrealistic cylinder radius is used, if compared to real dendrites size [88, 89].

The experiments performed by Dyrby et al. on post-mortem monkey data [90] and by Huang et al. on in-vivo human data [91] point out that estimates of axon diameter in WM are dependent on the gradient strength used for the acquisition and, in both studies, these estimates are seen to become more accurate as the b-value increases. The parameter Δ affects the estimated radius also. Diameter estimates obtained by Huang from data acquired at higher b-values (G > 145 mT m^−2^) are realistic, and close to estimates coming from histologic studies of the body of the corpus callosum. And they are also close to diameter for the “zeppelincylinder” model we used for the WM, although it has been estimated from datasets with a maximum b-value of G = 60 mT m^−2^[73]. Nonetheless, we remark that the other parameters we used for the zeppelincylinder model can be of limited accuracy. Recent studies [92] showed the advantage of using WM fiber dispersion models like [93, 94]. These models could be incorporated in the proposed framework to improve the quality of the phantom data.

The streamlines we used as “input connectome” may be a biased representation of the true brain connectivity diagram of the scanned volunteer. As a matter of fact, assessing the anatomical accuracy of results from any FT algorithm is currently a very challenging task. Fiber populations crossing at small angles [33] may have been not resolved. Additionally, CSD provides a high angular resolution, but it is not able to make distictions between crossing and fanning fibers inside a voxel, and it is challenged by fibers that follow narrow U-shaped patterns. Nevertheless, the pipeline we used reduces tractography biases, and iFOD2 allows an improved estimation in regions with curve bundles. The tractography technique we used produces tractograms that show a good intra-scan and inter-scan stability [86]. Besides, at voxel level, fiber densities are biologically meaningful [25], and this convinced us to introduce Eq 3 in our method.

A possible remark is that “fiber counting” is considered controversial as an analysis method [95]. We recall that the FT algorithm used for the input tractogram is designed to establish a relationship between the features of the estimated connectome and the ones of the DW MR images, minimizing many known biases.

## Conclusions

This paper proposes a method to generate phantom DW MRI data. The phantom consists of DW MRI data generated from WM streamlines estimated from real data. What sets our method apart is that the bundles are arranged with a level of geometric complexity comparable to what is expected in human WM, and the other brain tissues are included in our model as well. We included state-of-the-art attenuation models in order to get the most accurate “brain-like” DW MR phantom, and we made our phantom fully tunable in terms of simulated acquisition on a virtual MRI scanner. We showed that fiber bundles estimated with FT techniques exhibit a 3D structure similar to the one obtained by tractography on real datasets. We showed that the FA maps and fODFs estimated from such a phantom have a realistic sensitivity with respect to noise and other acquisition parameters. This is achieved without the need of human intervention. Additionally, we provided a framework within which connectivity oriented DW MRI methods validation is possible, and the features of the estimated connectomes can be investigated while many acquisition parameters are varied.
